# Clinical practice guidelines for translating pharmacogenomic knowledge to bedside. Focus on anticancer drugs

**DOI:** 10.3389/fphar.2014.00188

**Published:** 2014-08-19

**Authors:** José A. G. Agúndez, Gara Esguevillas, Gemma Amo, Elena García-Martín

**Affiliations:** ^1^Department of Pharmacology, University of ExtremaduraCáceres, Spain; ^2^ISCIII Research Network of Adverse Reactions to Allergens and DrugsMadrid, Spain

**Keywords:** biomarkers, adverse drug reactions, pharmacogenomics, clinical recommendations, clinical relevance

## Abstract

The development of clinical practice recommendations or guidelines for the clinical use of pharmacogenomics data is an essential issue for improving drug therapy, particularly for drugs with high toxicity and/or narrow therapeutic index such as anticancer drugs. Although pharmacogenomic-based recommendations have been formulated for over 40 anticancer drugs, the number of clinical practice guidelines available is very low. The guidelines already published indicate that pharmacogenomic testing is useful for patient selection, but final dosing adjustment should be carried out on the basis of clinical or analytical parameters rather than on pharmacogenomic information. Patient selection may seem a modest objective, but it constitutes a crucial improvement with regard to the pre-pharmacogenomics situation and it saves patients’ lives. However, we should not overstate the current power of pharmacogenomics. At present the pharmacogenomics of anticancer drugs is not sufficiently developed for dose adjustments based on pharmacogenomics only, and no current guidelines recommend such adjustments without considering clinical and/or analytical parameters. This objective, if ever attained, would require the use of available guidelines, further implementation with clinical feedback, plus a combination of genomics and phenomics knowledge.

Adverse drug reactions (ADRs) constitute a major problem in clinical practice, as these reactions may result in patient risk and/or therapeutic failure. The identification, validation, refinement, and clinical use of biomarkers of drug response are primary objectives in medical research. In this context, pharmacogenomic biomarkers have shown success as predictors of drug response and ADRs, though several limitations and barriers hamper the widespread clinical use of pharmacogenomic biomarkers. These factors have been analyzed in detail elsewhere (Deverka et al., 2007; Haga and Burke, 2008; Agundez, 2009; Agundez et al., 2009; Relling et al., 2010; Fernandez et al., 2012; Agúndez and García-Martín, 2014; Caudle et al., 2014; Quinones et al., 2014; Rossolatos and Aitchison, 2014).

Because anticancer drugs usually show high toxicity and/or narrow therapeutic index, the potential of pharmacogenomic biomarkers in anticancer therapy is particularly high. Pharmacogenomic biomarkers can be used in anticancer therapy for drug selection according to patient’s and/or tumor genomics, and it is expected that – often combined with therapeutic drug monitoring – it will constitute a powerful combination to optimize drug dosing. Some potentially relevant pharmacogenomic biomarkers for anticancer drugs which already have clinical recommendations are summarized in **Table 1**. In this perspective manuscript we briefly present our viewpoint on the potential of pharmacogenomics in the clinical use of anticancer drugs, as well as some personal insight and opinion on this issue.

**Table 1:** Examples of anticancer drugs with pharmacogenomic recommendations.

Drug	Drug Bank Accession No.	Gene/marker
Afatinib	DB08916	*EGFR*
Arsenic trioxide	DB01169	*PML/RARα*
Azathioprine	DB00993	*TPMT*
Bosutinib	DB06616	BRC/ABL1 Philadelphia chromosome
Brentuximab vedotin	DB08870	*CD30*
Busulfan	DB01008	Philadelphia chromosome
Capecitabine	DB01101	*DPYD*
Cetuximab	DB00002	*EGFR, KRAS*
Cisplatin	DB00515	*TPMT*
Crizotinib	Not available	*ALK*
Dabrafenib	DB08912	*G6PD, BRAF*
Dasatinib	DB01254	Philadelphia chromosome
Denileukin diftitox	DB00004	*ILR2A*
Erlotinib	DB00530	*EGFR*
Everolimus	DB01590	*ERBB2, ESR1*
Exemestane	DB00990	*ESR1*
Fluorouracil	DB00544	*DPD*
Fulvestrant	DB00947	ER receptor
Gefitinib	DB00317	*EGFR, CYP2D6*
Homoharringtonine	DB04865	*BCR/ABL1*
Ibritumomab	DB00078	*MS4A1*
Imatinib	DB00619	*KIT, BRC/ABL1, PDGFBR, FIP1L1/PDGFRA*
Irinotecan	DB00762	*UGT1A1*
Lapatinib	DB01259	*ERBB2*
Lenalidomide	DB00480	5q Chromosome deletion
Letrozole	DB01006	*ESR1, PGR*
Mercaptopurine	DB01033	*TPMT*
Nilotinib	DB04868	Philadelphia chromosome, *UGT1A1*
Panitumumab	DB01269	*EGFR, KRAS*
Pazopanib	DB06589	*UGT1A1*
Pertuzumab	DB06366	*ERBB2*
Ponatinib	DB08901	*BCR/ABL1*
Rasburicase	DB00049	*G6PD*
Rituximab	DB00073	*MS4A1*
Tamoxifen	DB00675	*ESR1, PGR, F2, F5*
Tegafur	Not available	*DPD*
Thioguanine	DB00352	*TPMT*
Tositumomab	DB00081	*MS4A1*
Trametinib	DB08911	*BRAF*
Trastuzumab	DB00072	*ERBB2*
Tretinoin	DB00755	*PML/RARα*
Vemurafenib	DB08881	*BRAF*

*Compiled from Agundez et al. (2012a,b), Caudle et al. (2013, 2014), and Relling et al. (2013), (http://www.fda.gov/drugs/scienceresearch/researchareas/pharmaco genetics/ucm083378.htm), (http://www.pharmgkb.org/page/cpicGeneDrugPairs) and CPIC Gene-Drug Pairs (see http://www.pharmgkb.org/contributors/consortia/cpic_gene-drug_pairs.jsp).*

The development of clinical guidelines for implementing pharmacogenomics knowledge in anticancer therapy is still at an early stage. Although **Table 1** shows over 40 anticancer drugs that have practice recommendations, clinical guidelines have been developed for a very limited number of these. These clinical guidelines, which are essential to implementing the use of pharmacogenomics (Agundez et al., 2012a,b; Crews et al., 2012; Caudle et al., 2013, 2014; Relling et al., 2013; Quinones et al., 2014), are being drafted for several gene/drug pairs. The Clinical Pharmacogenetics Implementation Consortium (CPIC), the Pharmacogenomics Knowledge Base (PharmGKB^1^, or the St. Jude Children’s Research Hospital^2^ as well as other international initiatives, are particularly active in providing guidelines and therapeutic recommendations based on pharmacogenomic testing.

Pharmacogenomic information may be used in clinical practice at diverse levels: the most conservative pharmacogenomics views aim to stratify patient populations (patient selection biomarkers) into those who should or should not receive a given drug (Green and Guyer, 2011). One step further is the use of pharmacogenomic information as a biomarker of clinical response, with drug dose adjustment based on pharmacogenomics tests [see for instance (Swen et al., 2011)]. So far, most guidelines aiming to adjust dose on the basis of pharmacogenomics information also recommend therapeutic drug monitoring and/or close surveillance of the clinical evolution of the patients based, for instance, on analytical biomarkers. A third step would be to substitute therapeutic drug monitoring for pharmacogenomic information. Further steps substituting, for instance, data on clinical evolution for pharmacogenomic data seem unrealistic.

Our personal opinion is that for anticancer drugs pharmacogenomics is as yet insufficiently developed to go beyond the first step, that is, pharmacogenomics for anticancer drug therapy should be limited to predicting whether individuals would respond to a determined drug, or the odds are that a patient will experience adverse effects with a determined drug. Accordingly, CPIC guidelines on anticancer drugs are rather conservative. These guidelines are based on the genetic status for loss of function gene variants, making different (but somewhat overlapping) recommendations for homozygous or heterozygous carriers of defect genes.

For azathioprine and *TPMT*, for example, the guidelines recommend using alternative drugs for homozygous individuals, or decreasing starting doses, and then tritrating for drug tolerance allowing 2–4 weeks to reach steady-state after each dose adjustment in heterozygous individuals (Relling et al., 2011, 2013). For mercaptopurine and *TPMT*, recommendations are to consider different drugs or to reduce drastically starting doses for homozygous individuals and to adjust doses based on myelosuppression and disease-specific guidelines, both in homozygous and heterozygous individuals (Relling et al., 2011, 2013). For thioguanine and *TPMT,* recommendations are to consider other drugs or to start with drastically reduced doses and to adjust doses based on myelosuppression and disease-specific guidelines, both in homozygous and heterozygous individuals (Relling et al., 2011, 2013). For capecitabine, fluorouracil, tegafur and *DPYD*, recommendations are to consider alternative drugs for homozygous individuals, or to reduce by about a 50% the starting dose and to adjust doses based on the toxicity or the pharmacokinetics [see for instance Caudle et al. (2013) or ].

It should be noted that these recommendations usually fall within one of two categories: to select an alternative drug or to reduce the starting dose by a determined percentage and then titrate the dosage according to the patient’s response. Drug adjustments therefore are not based on pharmacogenomic testing as such. All recommended changes in drug dosage should be supported by clinical or analytical biomarkers. We do not think that dosage recommendations should go beyond this point, that is, at the present stage of pharmacogenomic knowledge the use of clinical or analytical biomarkers should not be substituted by pharmacogenomic testing. The objective of dose adjustment based on pharmacogenomics tests only, if ever reached, would require further implementation of phenotype or pharmacokinetic inference and this would require a combination of genomics and phenomics knowledge at a level that we do not presently have.

When incorporating pharmacogenomics testing into routine clinical practice, we should bear in mind that the genotype is a surrogate biomarker of the metabolic status or the clinical response of a determined individual, with a determined drug, at a determined dose, and in a particular situation. Many variables influence the genotype–phenotype, gene-concentration and gene-dose relationships. For instance, gene variations very often do not cause relevant changes in drug plasma concentration. Another issue is that conventional pharmacogenomic genotyping classify alleles as functional by exclusion, that is, when no common enzyme inactivating mutations are identified. Unless patients are genotyped by using whole gene sequencing, the chances of mistyping are relatively high. Moreover, the analysis of some SNPs may yield ambiguous genotypes making it necessary to use haplotype reconstruction algorithms or diplotype–phenotype conversion tables (Agundez et al., 2008). Another issue is ethnic variability because commonly used genotyping tests may not be well suited to all human populations (Roco et al., 2012; Moreno-Guerrero et al., 2013).

Additional uncertainty in phenotype inference comes from the occurrence of induction and inhibition processes that may modify *in vivo* activity regardless of the genotype. Moreover, conventional genotyping misses the influence of other gene variations. Even taking all these issues into consideration it is clear that phenotypes may change as a result of several factors including environmental conditions, disease progression, concomitant drug therapy and many other phenomic factors (Houle et al., 2010; Neuraz et al., 2013). So far we are aware of some examples, probably the tip of the iceberg, of elements which can modify genotype–phenotype associations.

When developing guidelines for the clinical use of pharmacogenomic information for the use of anticancer drugs, these factors should be addressed in the guidelines, bearing in mind that the combination of genotyping, drug pharmacokinetics, pharmacodynamics, and phenome-wide studies in large groups of patients with different clinical situations is necessary in order to gain more ground in genotype–phenotype association. Recently the CPIC group released the development process of guidelines designed to provide guidance to clinicians as to how available genetic test results should be interpreted to ultimately improve drug therapy (Caudle et al., 2014). These guidelines are based on the assessment of a known gene–drug relationships, the identification of content experts and the formation of writing committee, retrieval, summarization and presentation of the evidence linking genotype to drug variability, development of therapeutic recommendation and assignment of strength of the recommendation, internal and external review, and periodic review and guideline updates. A crucial point in the development of guidelines for the clinical use of pharmacogenomics information includes caveats to the effect that non-genetic considerations are also important for prescribing decisions. Thiopurine methyltransferase (TPMT) is a good example of this. Whereas a strong phenotype/genotype association exists for *TPTM* (Ford et al., 2009) most of the azathioprine/mercaptopurine-induced adverse reactions and the efficacy of therapy are not explained by *TPMT* polymorphisms (Palmieri et al., 2007).

Besides known and extensively discussed barriers and limitations to implementing the use of pharmacogenomic testing, related to institutional support or clinician’s awareness of the usefulness of pharmacogenomics testing, there an important barrier which is attributable to the scientific community, that is, some possible overstating of our findings or overestimating the current power of pharmacogenomics. We believe that one of the best services we can give to pharmacogenomic testing is to recognize its limitations and to work to solve them. In our opinion, part of the disenchantment experienced with pharmacogenomics in recent years may be at least partly attributable to an overoptimistic expectation of making a safe and reliable personalized dose adjustment based on pharmacogenomics tests only. We still have a long way to go before we can do that. In the meantime, the use of pharmacogenomic guidelines such as those already published by CPIC^3^ and the assessment of their performance in clinical practice would provide a highly valuable feedback that could be used to refine the whole process and eventually to gain more ground on the inference of drug response.

## Conflict of Interest Statement

The Guest Associate Editor Luis Quinones declares that, despite having published together with the authors Elena García-Martín and José A. G. Agúndez in the past 2 years, the review process was handled objectively. The authors declare that the research was conducted in the absence of any commercial or financial relationships that could be construed as a potential conflict of interest.

## Footnotes

1 http://www.pharmgkb.org

2 http://www.stjude.org/pg4kds

3 https://www.pharmgkb.org/page/cpicGeneDrugPairs
